# High Expression of PAPP‐A Predicts Poor Outcomes in Oestrogen Receptor‐Positive Breast Cancer Patients

**DOI:** 10.1002/cam4.71815

**Published:** 2026-04-13

**Authors:** Zeanap Mabruk, Esme Bullock, Xue Xiao, Jingjing Guo, Xuan Zhu, Laura Gómez‐Cuadrado, Claus Oxvig, Elizabeth Mallon, Aula Ammar, Amna Malty, Kathryn Pennel, Joanne Edwards, Valerie G. Brunton

**Affiliations:** ^1^ Cancer Research UK Scotland Centre (Edinburgh), Institute of Genetics & Cancer University of Edinburgh Edinburgh UK; ^2^ Department of Pathology, Sichuan Provincial People's Hospital, School of Medicine University of Electronic Science and Technology of China Chengdu China; ^3^ Department of Molecular Biology and Genetics University of Aarhus Aarhus Denmark; ^4^ Department of Pathology Queen Elizabeth University Hospital Glasgow UK; ^5^ School of Cancer Sciences, Wolfson Wohl Cancer Research Centre University of Glasgow Glasgow UK

**Keywords:** breast cancer, IGF‐1 signalling, invasive lobular carcinoma, pregnancy‐associated plasma protein‐A

## Abstract

**Introduction:**

Insulin‐like growth factor 1 (IGF‐1)/IGF‐1 receptor (IGF‐1R) signalling is activated in breast cancer and associated with disease progression. Pregnancy‐associated plasma protein‐A (PAPP‐A) is a metalloproteinase that can cleave IGF binding proteins leading to the release of bioactive IGF‐1 and the subsequent activation of IGF‐1 signalling. Here, we aimed to assess the prognostic significance of PAPP‐A in breast cancer.

**Methods:**

Breast cancer tissue microarrays were stained for PAPP‐A and expression correlated with survival and other clinical features. Analysis of publicly available data sets was carried out to determine associations between *PAPPA* and gene sets associated with IGF‐1/IGF‐1R pathway activation.

**Results:**

PAPP‐A was expressed in both the tumour and stromal compartments in breast cancer specimens and was higher in oestrogen receptor (ER)‐negative (ER‐) than ER‐positive (ER+) cases. There was a significant association between high PAPP‐A expression and reduced cancer‐specific survival (CSS) in ER+ (HR 1.389, 95% CI 1.051–1.836, *p* = 0.021) but not ER‐ patients (HR 1.040, 95% CI 0.712–1.598, *p* = 0.838). In a second cohort of ER+ invasive lobular carcinoma (ILC) PAPP‐A expression was also associated with reduced CSS (HR 1.765, 95% CI 1.098–2.836, *p* = 0.019). *PAPPA* correlated with REACTOME PI3K‐AKT and IGF1R signalling gene sets in ER+ breast cancers.

**Conclusion:**

High expression of PAPP‐A is associated with poor prognosis in ER+ breast cancer and correlates with IGF‐1/IGF‐1R pathway activation.

## 1 Introduction

Insulin‐like growth factor 1 (IGF‐1) signalling is a complex and highly regulated pathway that plays a crucial role in mammary gland development and also in the initiation and progression of breast cancer [1, 2, 3]. Binding of IGF‐1 to its cognate receptor IGF‐1R triggers its autophosphorylation and activation, leading to downstream activation of signalling pathways via the insulin receptor substrate 1 (IRS‐1). The two predominant IGF‐1 responsive pathways are the phosphatidylinositol 3‐kinase/AKT kinase (PI3K/AKT) pathway and the RAF kinase/mitogen activated protein kinase (RAF/MAPK) pathway, which can both provide proliferative and survival signals [1, 2].

Several studies have shown strong associations between IGF‐1R pathway activation and clinical outcomes in breast cancer patients and other tumour types [4, 5, 6], which has led to the development of strategies to target IGF‐1/IGF‐1R signalling pathways. These include anti‐IGF1 and IGF‐1R antibodies, and dual insulin receptor (IR)/IGF‐1R tyrosine kinase inhibitors [7, 8]. Although these have been trialled extensively in a range of tumour types, including breast cancer, the results have been disappointing, either due to a lack of efficacy resulting from activation of compensatory signalling pathways or metabolic toxicity linked to systemic effects on insulin secretion. In addition, a greater understanding of how IGF‐1R signalling is regulated will help to identify which patients may benefit from different targeting strategies. For example, the availability of bioactive IGF‐1 is controlled by its binding to IGF binding proteins (IGFBPs), and pregnancy‐associated plasma protein‐A (PAPP‐A), a metalloproteinase that can cleave IGFBPs, and this plays an important role in IGF‐1 signalling [9]. PAPP‐A is active when bound to glycosaminoglycans on the cell surface and can activate transmembrane IGF‐1Rs in close proximity through the release of localised concentrations of free IGFs [10]. For this reason, attempts have been made to develop strategies to inhibit PAPP‐A [11], the benefit over other IGF‐1/IGF‐1R targeted approaches which can have effects on insulin receptor signalling leading to hyperglycaemia adverse effects [12], being that inhibition of IGF‐1 signalling would be restricted to tissues in which PAPP‐A was active. Indeed, preclinical in vivo studies have shown anti‐tumour activity of an anti‐PAPP‐A blocking antibody in lung, ovarian and sarcoma models [13, 14, 15].

The role of PAPP‐A in breast cancer has been recently reviewed [16], and although reports on the association between *PAPPA* and survival differ, this may reflect differences between breast cancer subtypes [17, 18]. PAPP‐A protein expression has been reported in breast cancer tissue and there is also an increased expression in pregnancy‐related breast cancer compared with normal breast tissue [18, 19, 20, 21]. However, PAPP‐A promoter methylation associated with down regulation of PAPP‐A expression has also been described in some breast cancers [19]. These studies were carried out on a limited number of tumours and a more comprehensive analysis of PAPP‐A in breast cancer tissues is required to further our understanding of its relevance and importance to disease progression.

Here we set out to determine whether PAPP‐A has a prognostic role in breast cancer using a large retrospective cohort of breast cancer patients. We also assessed the significance of PAPP‐A in invasive lobular carcinoma (ILC) where we previously identified *PAPPA* as a stromal enriched gene that was secreted from cancer‐associated fibroblasts (CAFs) in the tumour microenvironment [17].

## 2 Methods

### 2.1 Patient Samples

Details of the tissue microarray generated in Glasgow from a retrospective cohort of 850 breast cancer tumours has been described previously [22]. An additional cohort of 246 human primary operable ILC tumours was collected under the approval of the research ethics committee of the West of Scotland Research Ethics Service REC4 (REC reference: 22/WS/0020, IRAS project ID: 306447), and a cohort of ER+ breast cancers were retrieved from Sichuan Provincial People's hospital (Chengdu, China) after approval by the hospital's ethics committee. Further details in Supporting Information.

### 2.2 Immunohistochemistry

PAPP‐A staining was performed on sections from FFPE tumour blocks. Slides were incubated at 50°C for 40 min following deparaffinisation by xylene and rehydration by a series of descending concentrations of alcohol. After heat‐induced antigen retrieval, slides were blocked with endogenous peroxidase‐blocking solution (Dako, S2023) and then serum‐free protein block (Dako, X0909). Slides were incubated at 4°C overnight with PAPP‐A antibody (Novus Biologicals, #NBP1‐90087, 1:50), and after incubation with secondary antibody (EnVision+HRP #K4011, Dako) and DAB development, slides were counterstained with haematoxylin. Slides were scanned using a Hamamatsu NanoZoomer and viewed on NDP Viewer. Protein expression levels were quantified by weighted histoscore in tumour and stromal compartments as described previously [22].

### 2.3 Publicly Available Data Sets

All analysis was restricted to ER+ ILC and ER+ no special subtype (NST) samples. TCGA Firehose RNASeq RSEM and clinical data were accessed through cbioportal in June 2022. TCGA Firehose RPPA level 4 median normalised and batch corrected data was downloaded from https://tcpaportal.org/tcpa/download.html in April 2020. ESTIMATE stromal scores were accessed in October 2021 from https://bioinformatics.mdanderson.org/estimate/disease.html.

### 2.4 Statistical Analysis

To set threshold values for high and low expression of each protein, log‐rank statistics were performed in R Studio (2024.12.0 + 467) using survminer, tidyverse, survival and maxstat packages. All further statistical analyses of tissue‐based studies were done using IBM SPSS version 29 (IBM, New York, USA). Chi‐squared testing was carried out to assess the association between protein expression and the patient clinicopathological characteristics. Kaplan–Meier log‐rank curves were used to study associations between protein expression and cancer‐specific survival (CSS). Cox regression survival analysis was performed for each variable of interest and other prognostic factors and recorded as a hazard ratio with a 95% confidence interval (CI). Statistically significant variables (*p* < 0.05) on univariate analysis were entered into the multivariate analysis using backward conditional models. Non‐parametric Mann–Whitney test and Kruskal–Wallis followed by Dunn's test were used to assess the association between discrete protein expression groups and continuous variables of interest, and bar charts were plotted using SPSS. Single sample gene set variation analysis (GSVA) was carried out using the GSVA R package (v2.0.6). A Shapiro–Wilk test was performed to assess normality of gene expression and GSVA scores and a Spearman's correlation of gene expression and GSVA scores was carried out using GraphPad Prism v10.4.2. For all analyses, statistical significance was represented as *p* < 0.05.

## 3 Results

### 3.1 PAPP‐A Expression in Breast Cancer

**FIGURE 1:**
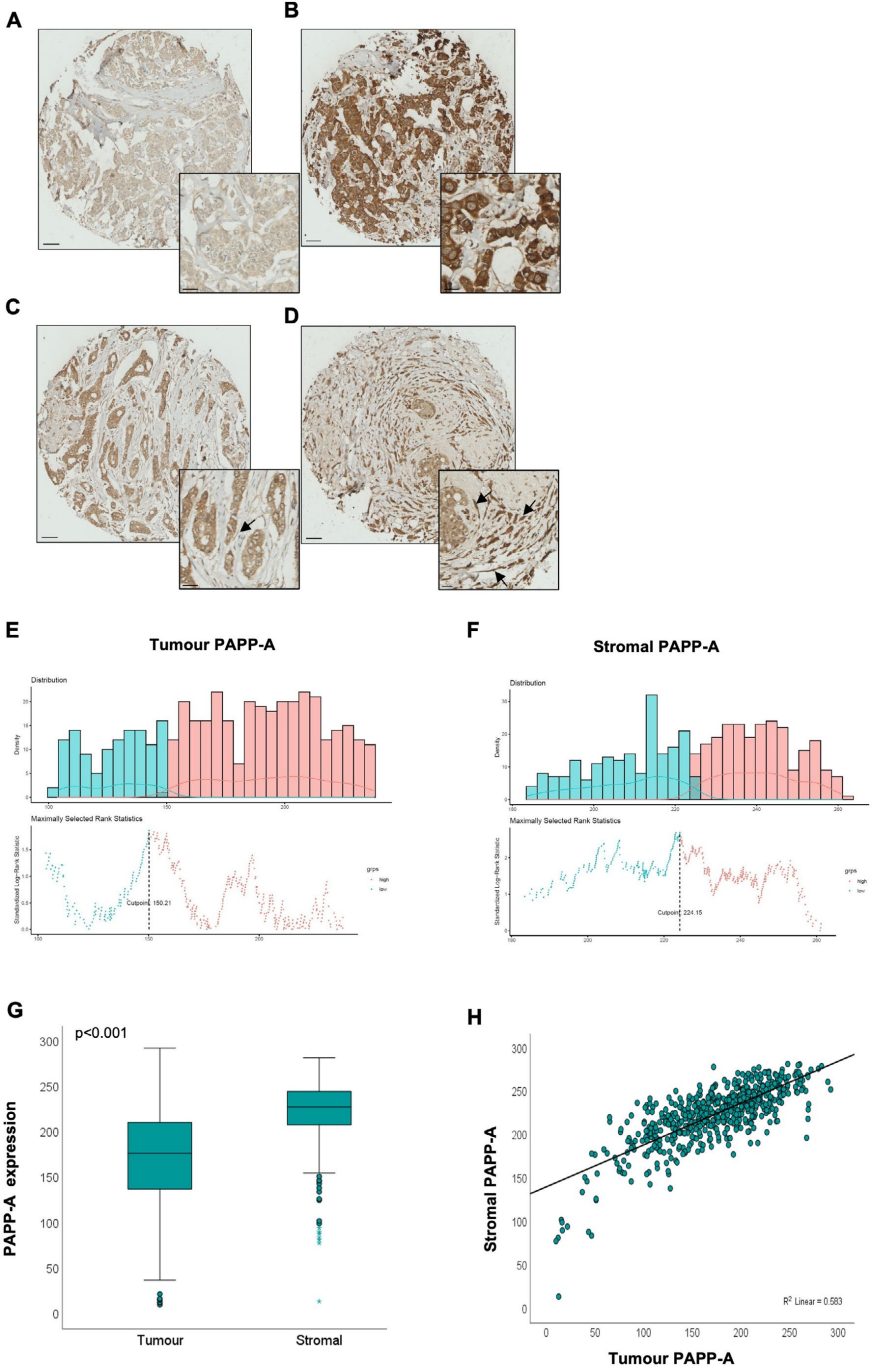
Expression of PAPP‐A in breast cancer. (A‐D) Representative images of PAPP‐A staining showing predominantly weak cytoplasmic staining (A), strong cytoplasmic staining (B), weak stromal staining (C), and predominantly strong stromal staining (D). Scale bars, 50 μm with an inset of 20 μm. (E, F) Density and scatter plots for visualisation of optimal cut‐off points for high and low expression of (E) tumour PAPP‐A and (F) stromal PAPP‐A. (G) Boxplots to illustrate subcellular expression of PAPP‐A in tumour and stromal compartments. Mann–Whitney test *p* < 0.001. (H) Scatter plot to display positive correlation between expression of tumour and stromal PAPP‐A. Pearson correlation coefficient = 0.764, *p* < 0.001.

**FIGURE 2:**
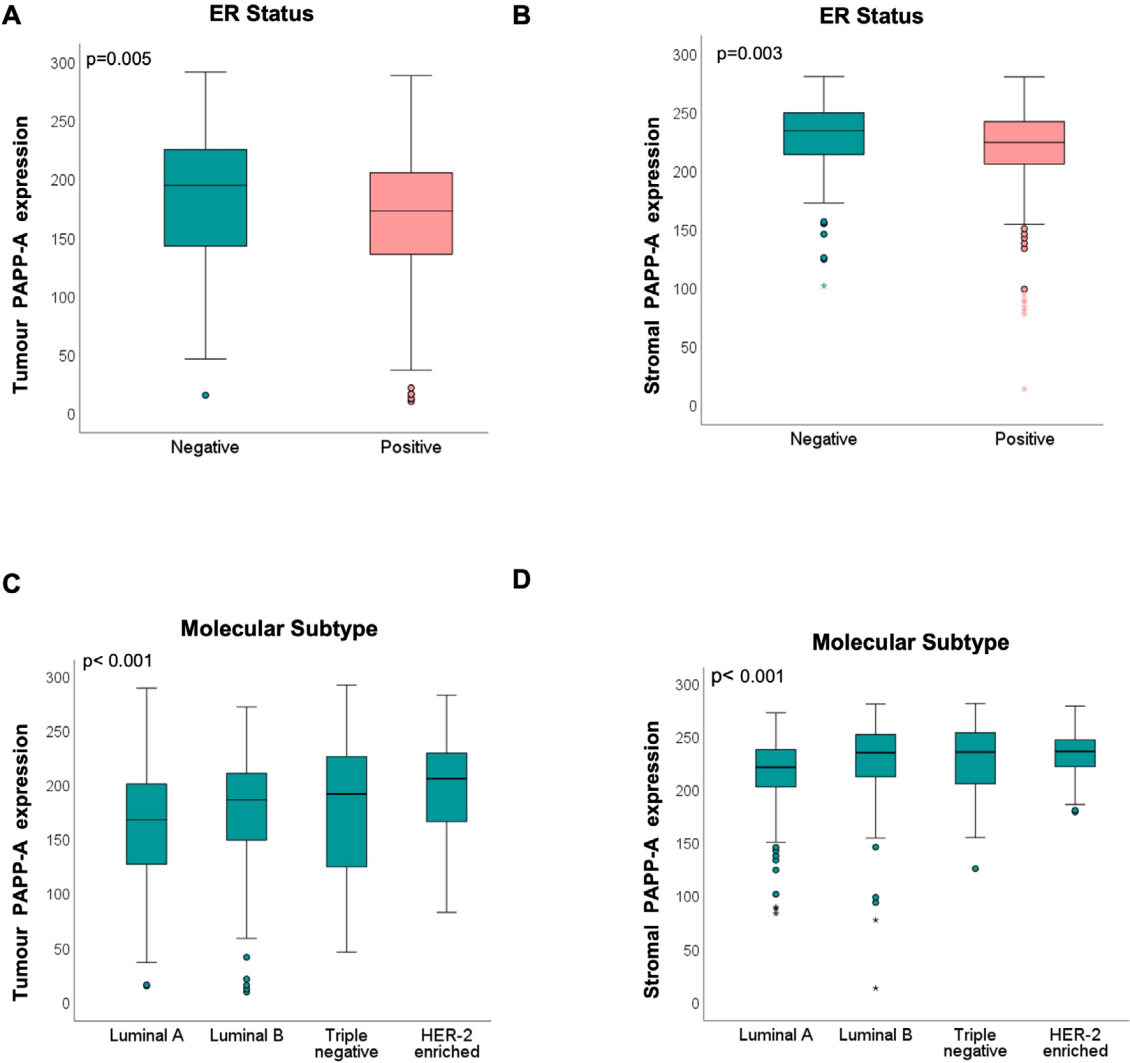
Differential expression of PAPP‐A in the tumour and stroma of breast cancer sub‐types. (A, B) Boxplots to illustrate the differences in tumour and stromal PAPP‐A expression in ER‐negative and ER‐positive tumours and (C, D) in different molecular subtypes. Mann–Whitney test (A, B) and Kruskal–Wallis and Dunn‐Bonferroni post hoc testing (C, D) carried out to compare PAPP‐A expression.

Immunohistochemical analysis of PAPP‐A was carried out on a tissue microarray of 573 breast cancers (Figure S1). PAPP‐A expression was observed both in the tumour and stroma (Figure 1A–D). An optimal cut‐off point of 150.21 for tumour expression and 224.15 for stromal expression was determined to stratify tumours into high or low expression (Figure 1E,F). PAPP‐A expression was higher in the stroma with a mean histoscore of 223.18, while the tumour PAPP‐A showed a mean histoscore of 172.46 (*p* < 0.001) (Figure 1G). There was also a significant positive correlation between tumour and stromal PAPP‐A expression (Figure 1H). Compared with oestrogen receptor (ER)‐positive cases, tumour and stromal PAPP‐A were expressed at a higher level in ER‐negative tumours (Figure 2A,B). There was a significant difference for both tumour and stromal PAPP‐A expression across the molecular subtypes of breast cancer (*p* < 0.001), with triple‐negative and HER2‐enriched tumours displaying higher expression levels of tumour and stromal PAPP‐A compared with the luminal A molecular subtype (*p* < 0.001), while luminal B exhibited lower tumour PAPP‐A levels compared with the HER2‐enriched subtype (*p* = 0.025) (Figure 2C,D).

**FIGURE 3:**
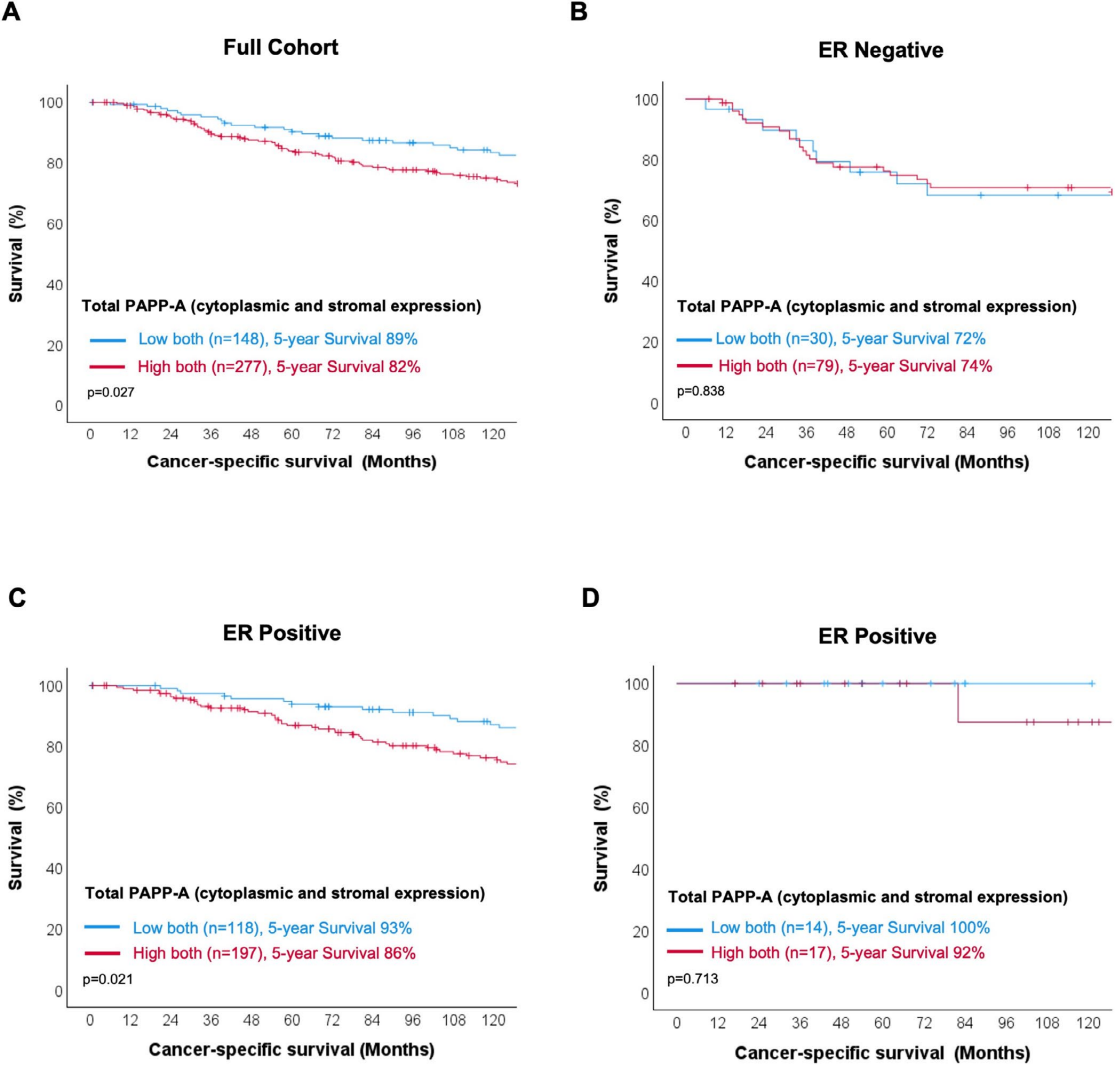
Association between total PAPP‐A expression and CSS. Kaplan Meier curves showing the association between total PAPP‐A expression and CSS across the full Glasgow cohort (A), in ER‐negative (B), and ER‐positive patients (C), and ER‐positive patients in the Chengdu cohort (D).

Subsequently, the association between PAPP‐A expression and CSS was assessed. Within the total cohort, patients with combined high tumour and high stromal (total) PAPP‐A expression were associated with a worse prognosis (HR 1.288, 95% CI 1.029–1.611, *p* = 0.027) (Figure 3A). When patients were subdivided by ER status there was a significant association between high total PAPP‐A expression and reduced CSS in ER‐positive (HR 1.389, 95% CI 1.051–1.836, *p* = 0.021) but not ER‐negative patients (HR 1.040, 95% CI 0.712–1.520, *p* = 0.838) (Figure 3B,C). Analysis of an additional cohort of 172 ER+ cancers showed a similar trend with increased total PAPP‐A associated with worse survival, although this did not reach statistical significance (HR 5.759, 95% CI NA, *p* = 0.713) (Figure 3D).

When the Glasgow cohort of 573 breast cancers was analysed by individual molecular subtypes, there was no notable correlation between total PAPP‐A expression and CSS in luminal A (HR 1.343, 95% CI 0.873–2.069, *p* = 0.180), luminal B (HR 1.334, 95% CI 0.905–1.965, *p* = 0.145), triple‐negative (HR 0.861, 95% CI 0.543–1.366, *p* = 0.525), or HER2‐enriched (HR 0.996, 95% CI 0.471–2.106, *p* = 0.991) (Figure S2). When analysing either tumour or stromal PAPP‐A expression across the whole cohort there was a significant association with stromal PAPP‐A and CSS, and although a similar trend was seen with tumour PAPP‐A this did not reach significance. In addition, high expression of both tumour and stromal PAPP‐A associated with worse survival in ER‐positive patients but not ER‐negative patients (Figure S3). Multivariate analysis using the Cox hazard model was then carried out for total and stromal PAPPA across the cohort. The tumour and stromal PAPP‐A expression was not independently associated with worse CSS (*p* = 0.166, *p* = 0.141, respectively). Among the other clinicopathological variables, patients with larger tumour size and positive lymph node status were independently associated with poorer outcomes (Tables S1 and S2). Chi‐squared analysis showed that high total PAPP‐A was significantly associated with tumours of higher grade (*p* < 0.001), ER, PR and HER2 status (*p* = 0.039, *p* = 0.016 and *p* < 0.00, respectively), and a high proliferation index Ki67 (*p* = 0.001) (Table S3). Patients with high expression levels of stromal PAPP‐A were associated with tumours of larger size (*p* = 0.016), higher grade (*p* < 0.001), ER‐negativity (*p* = 0.004), PR‐negativity (*p* = 0.003) and HER2 positivity (*p* = 0.003) (Table S4).

### 3.2 PAPP‐A Expression in ILC

**FIGURE 4:**
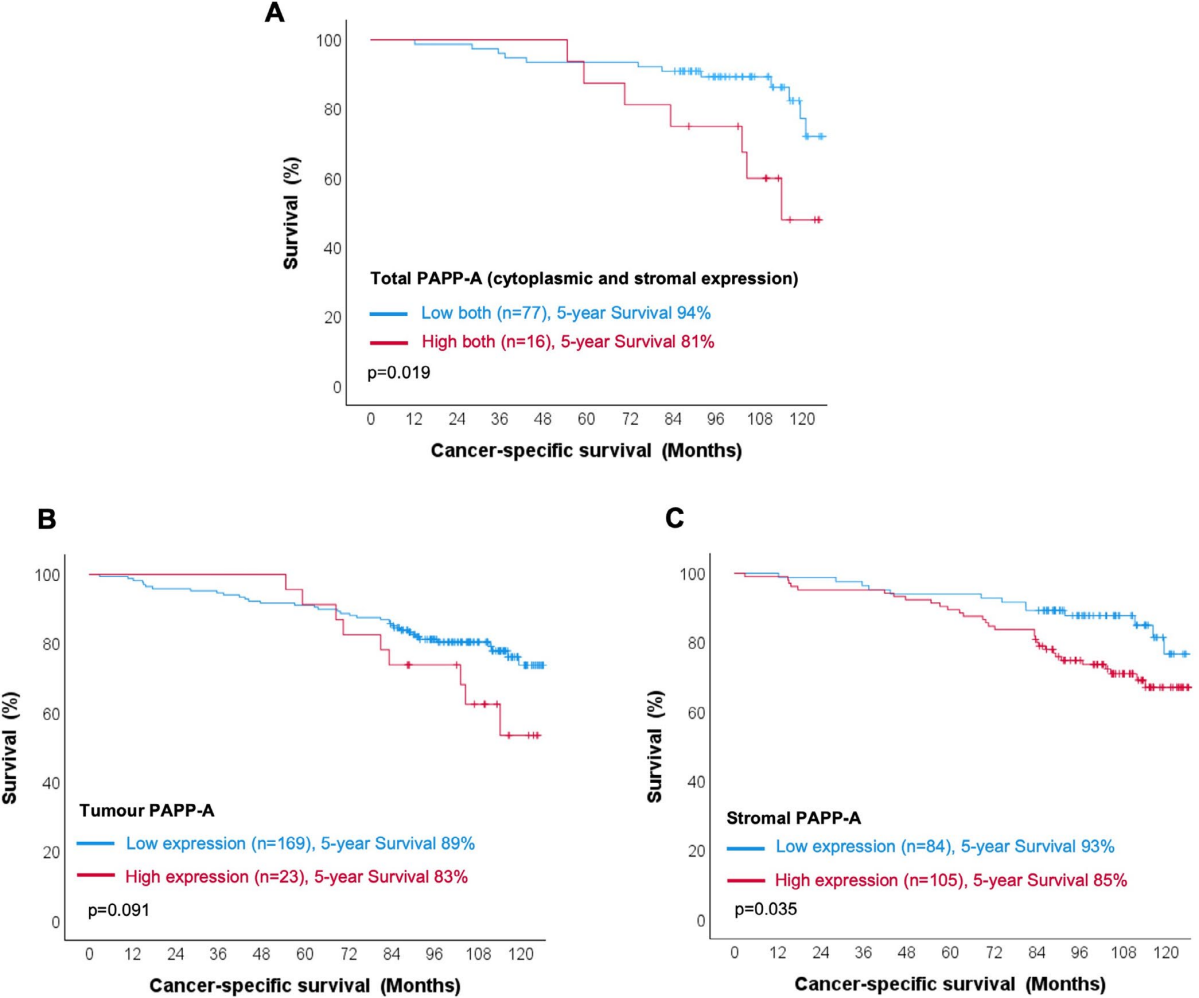
Association between total PAPP‐A expression and CSS in ILC. Kaplan Meier curves showing the association between total (A), tumour (B) and stromal (C) PAPP‐A expression and CSS.

ILC is a histological subtype of breast accounting for around 15% of breast cancer cases with more than 90% expressing ER [23]. ILC is defined by a loss of E‐cadherin which can sensitise cells to treatment with IGF‐1R, PI3K and AKT inhibitors, with ILC cell lines and patient‐derived models being sensitive to inhibitors targeting these pathways [24, 25, 26]. In addition, IGF‐1 pathway activity is enhanced in ILC compared to ER+ invasive breast cancer of no special type (NST), previously referred to as invasive ductal carcinoma [24, 25]. As we had previously identified *PAPPA* as a stromal enriched gene in ILC whose expression was increased in ILC compared with ER+ NST [17], we looked at PAPP‐A expression in a cohort of 246 human primary operable ILC. Across the cohort, an elevated expression of total PAPP‐A (combined high tumour and high cytoplasmic) was associated with a worse prognosis (HR 1.765, 95% CI 1.098–2.836, *p* = 0.019) (Figure 4A). When tumour and stromal PAPP‐A were individually analysed, a significant association between the high stromal PAPP‐A and reduced survival was found (HR 2.005, 95% CI 1.049–3.833, *p* = 0.035), whereas tumour PAPP‐A showed only a notable association with no statistical significance (HR 1.876, 95% CI 0.903–3.896, *p* = 0.091) (Figure 4B,C). On multivariate survival analysis using the Cox hazard model, total PAPP‐A and stromal PAPP‐A expression were not independently associated with worse CSS (*p* = 0.160, *p* = 0.087, respectively) (Tables S5 and S6).

### 3.3 *IGF1* and *PAPPA* Correlate With Stromal Content of Tumours

**FIGURE 5:**
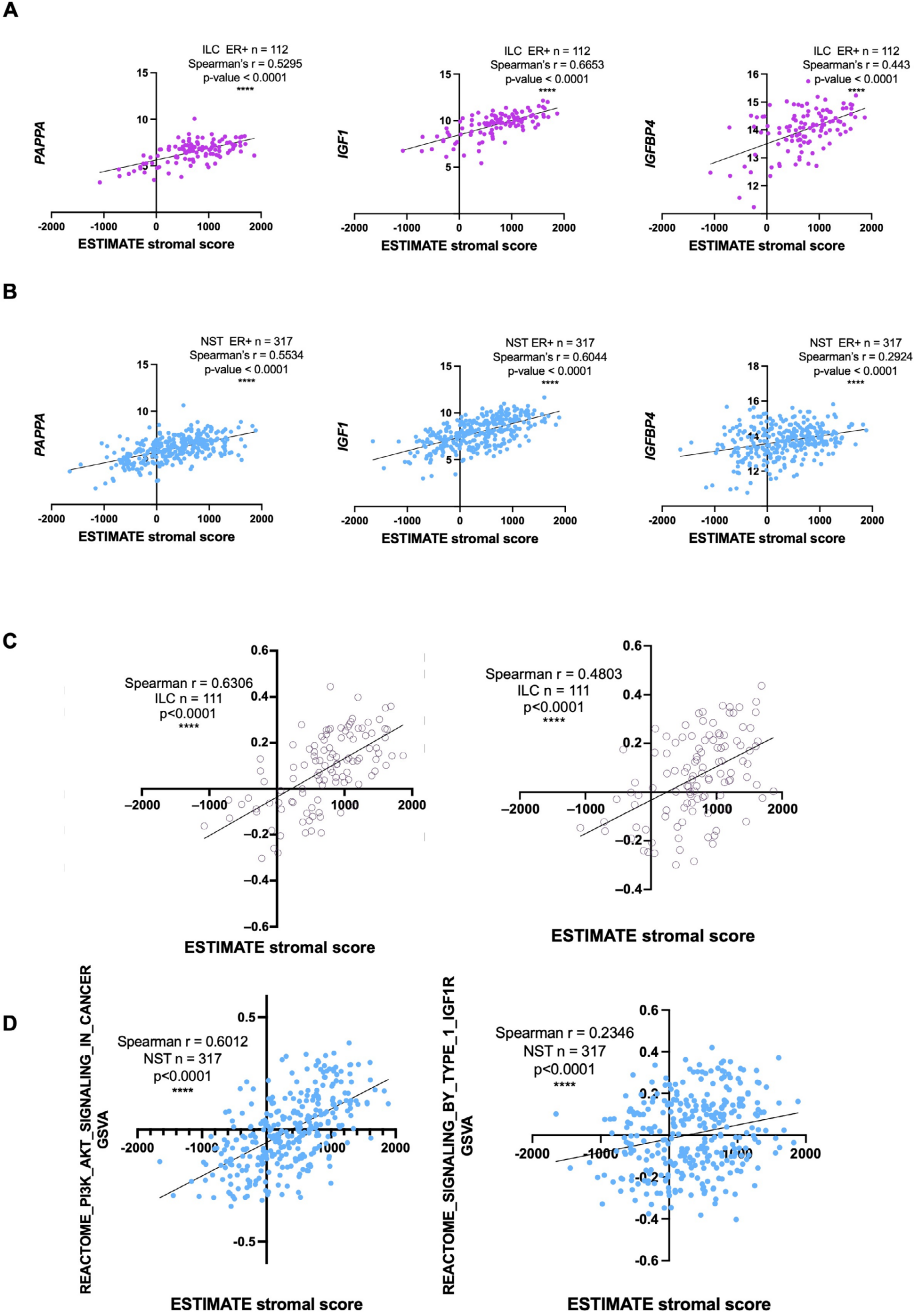
*IGF1* and *PAPPA* correlate with stromal content of tumours. Correlation of *PAPPA, IGF1* and *IGFBP4* with ESTIMATE stromal content in ER+ ILC (A) and ER+ NST tumours (B). Correlation of REACTOME PI3K_AKT and IGF1R signalling gene sets with ESTIMATE stromal content in ER+ ILC (C) and ER+ NST tumours (D). Spearman correlation carried out in GraphPad Prism.

As PAPP‐A expression was higher in the stroma we asked whether there was an association between PAPP‐A and the stromal content of ER+ breast cancer patients. Using ESTIMATE, which determines the stromal composition of bulk RNASeq data sets based on gene expression [27], analysis of TCGA RNASeq data showed a significant positive correlation between the ESTIMATE scores and *PAPPA* in both ER+ ILC and ER+ NST tumours (Figure 5A,B) consistent with our previous findings that PAPP‐A can be secreted from CAFs [17]. There was also a significant positive correlation between the ESTIMATE scores and *IGF1* in both ER+ ILC and ER+ NST tumours, and also *IGFBP4* which is exclusively cleaved by PAPP‐A [28], leading to increased bioavailability of IGF‐1 (Figure 5A,B). There was a significant positive correlation between the ESTIMATE scores and expression of REACTOME PI3K‐AKT and IGF‐1R signalling gene sets in both ER+ ILC and ER+ NST tumours, with the correlation between stromal content and IGF‐1R genes being much stronger in the ER+ ILC tumours (NST *n* = 317, Spearman r = 0.2346; ILC *n* = 111, Spearman r = 0.4803) (Figure 5C,D). Together, this demonstrates the importance of the tumour microenvironment in IGF‐1 pathway activation in both ER+ ILC and ER+ NST tumours, and the increased IGF‐1 pathway activation identified in ILC most likely reflects the higher stromal content reported in ILC tumours [29, 30].

### 3.4 PAPP‐A Association With IGF‐1 Pathway Activation

**FIGURE 6:**
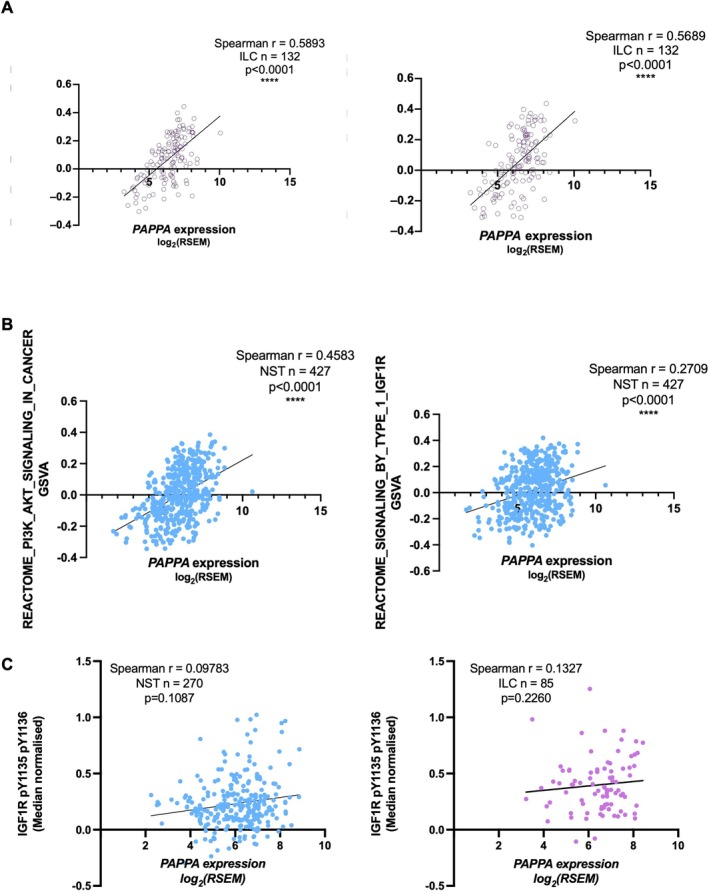
*PAPPA* correlates with IGF‐1R pathway activation. Correlation of *PAPPA* with REACTOME PI3K_AKT and IGF1R signalling gene sets in ER+ ILC (*n* = 132) (A) and ER+ NST (*n* = 427) tumours (B) and with pY1135/6 IGF‐1R in ER+ ILC (*n* = 85) and ER+ NST (*n* = 270) tumours from TCGA RPPA dataset (C). Spearman correlation carried out in GraphPad Prism.

As there was an association between PAPP‐A and CSS in ER+ breast cancer, we then asked whether *PAPPA* expression was associated with an increased IGF‐1 pathway activation. Analysis of the TCGA data using single sample GSEA showed a significant positive correlation between *PAPP‐A* and both REACTOME PI3K‐AKT and IGF1R signalling gene sets in ER+ NST and ER+ ILC tumours (Figure 6A,B). In the TCGA reverse phase protein array data set, there was also a significant positive correlation between *PAPP‐A* and pIGF1R in ER+ NST and ER+ ILC, although these did not reach significance (Figure 6C).

## 4 Discussion

This study represents the largest analysis to date of PAPP‐A expression across breast cancer subtypes. We show that PAPP‐A is expressed across all molecular subtypes, with luminal A patients having the lowest levels of PAPP‐A, while triple negative and HER‐2 enriched subtypes have the highest levels. Previously, analysis of TCGA breast cancer data set showed that *PAPPA* was significantly co‐expressed with epithelial‐to‐mesenchymal transition (EMT) markers, *VIM*, *TWIST1*, *SNAI1*, *SNAI2*, *ZEB1* and *ZEB2*, and the increased expression in triple negative and HER‐2 enriched tumours may reflect the mesenchymal phenotypes of these subtypes [31, 32]. Here we show across the whole cohort that PAPP‐A is associated with worse prognosis, and when broken down into subtypes, this is evident only in ER+ positive cancers. Although in a smaller cohort of patients this did not reach significance and may reflect the smaller sample size and/or different treatment regimens between the United Kingdom and China. The significance of PAPP‐A in ER+ breast cancer is not clear but may result from the well‐documented interaction between IGF‐1 and ER signalling pathways, where a reciprocal regulation of pathway activity and dependency has been identified [2]. Although PAPP‐A levels were higher in ER‐ tumours, there was no association with survival, suggesting that the interaction between the IGF‐1 and ER signalling pathways may be a more important driver of tumour progression than increased PAPP‐A alone. In another study of 45 predominantly triple negative breast cancers, there was also no correlation of PAPP‐A with overall survival, although the authors did note a trend towards worse survival and an association with high grade tumours and lymph node involvement [18]. Analysis of PAPP‐A and other IGF signalling‐associated proteins in serum showed that increased IGFBP‐2 and PAPP‐A were independently prognostic for recurrence‐free and overall survival, but PAPP‐A was not prognostic for early recurrence in breast cancer patients [33]. In addition, expression of a PAPP‐A‐driven genetic signature may be useful to identify breast cancer patients at higher risk of developing metastatic disease [34]. Together these results indicate that PAPP‐A is important in the progression of breast cancer and is further supported by studies which have identified a role for PAPP‐A in driving pro‐tumourigenic phenotypes, including proliferation, migration, invasion, EMT and tumour growth [18, 21]. In addition, overexpression of PAPP‐A in the mammary gland promotes tumourigenesis in parous mice [35]. Although these studies support a pro‐tumourigenic role for PAPP‐A, one study has reported that PAPP‐A protein loss is associated with delayed mitotic progression and increased invasiveness [19]. The authors suggest that PAPP‐A promoter methylation plays an important role early in the development of breast cancer and analysis of additional breast cancer cohorts will help to unravel this dichotomy.

PAPP‐A is known to be expressed and secreted by CAFs within the tumour‐associated stroma and this can lead to cleavage of IGFBP4 and activation of IGF‐1 signalling [17, 36, 37]. In hepatocellular cancer, stromal associated PAPP‐A can impact cancer‐promoting phenotypes [36]. Previously we identified *PAPPA* as a stromal enriched gene following laser microdissection of tumour and stroma in ER+ breast cancers [17] and here we show that PAPP‐A protein levels are also increased in the stroma compared with the tumour compartment and additionally show that increased *PAPPA* is correlated with the stromal content of ER+ tumours. Interestingly, only stromal PAPP‐A was associated with worse CSS, although the same trend was seen for tumour PAPP‐A. Taken together this supports an important role for paracrine PAPP‐A signalling pathways in ER+ breast cancer. The high stromal content of PAPP‐A in ER‐ breast cancers supports paracrine PAPP‐A signalling in these tumours as well. However, this was not associated with poor survival suggesting that it is not a major driver of ER‐ tumours.

The paracrine signalling axis may be of particular importance in ER+ ILC which are characterised by a high stromal content [29, 30] with both IGF‐1 and PAPP‐A being secreted by ILC‐derived CAFs [17, 29]. ER+ ILC are also known to have increased IGF‐1 pathway activation compared with ER+ NST [25, 26, 30]. This has been linked to the loss of the cell–cell adhesion protein E‐cadherin, the defining feature of ILC, via a mechanism that involves increased availability of IGF‐1R on the cell surface of E‐cadherin defective cells [24, 25, 26, 38]. The secretion of PAPP‐A would lead to an increased local bioavailability of IGF‐1 within the tumour microenvironment thereby further enhancing IGF‐1 pathway activation.

Attempts to target the IGF‐1R, including in ER+ breast cancer, have not been successful, showing limited benefit for patients [7, 8]. However, these studies lacked evidence of pathway activation with no biomarker driven stratification of patients. A number of biomarker strategies have been investigated, including intratumoural and serum levels of IGF‐1, expression of IGF‐1R and activated phosphorylated IGF1R, and expression of IGFBPs, but there have been inconsistencies when associating these with prognosis [1]. It is likely that a combination of markers will be required to provide prognostic information and provide a robust read‐out of pathway activation. However, it is important to note that in addition to its effects on IGF‐1 signalling, PAPP‐A has been reported to have additional effects that could impact breast cancer progression and survival. These include a reported role in immune evasion in Ewing's sarcoma [14] and remodelling of collagen during breast cancer development, where a combined PAPP‐A dependent gene set provided prognostic information on the development of metastases [34]. Furthermore PAPP‐A activity itself is tightly regulated by proteins that have also been identified as prognostic markers in breast cancer. These include the stanniocalcins STC1 and STC2, which are secreted polypeptides that bind to PAPP‐A and potently inhibit its catalytic function [39]. It will be important to consider these markers going forward and their relationship to PAPP‐A in ER+ breast cancer. A better understanding of these interactions could also provide additional support for direct targeting of PAPP‐A. Studies in mice have shown that expression of an IGFBP4 protein that is resistant to PAPP‐A‐dependent protease cleavage inhibits tumour growth and metastasis in breast cancer models [40, 41], while the use of an inhibitory PAPP‐A antibody can reduce tumour growth in models of lung, ovarian and sarcoma [13, 14, 15] suggesting that this may be a viable approach for targeting PAPP‐A.

## Author Contributions

V.G.B., Z.M., L.G.‐C., C.O. conceptualised the project. Z.M., E.M., K.P., J.E. curated and analysed the Glasgow cohorts. X.X, J.G, X.Z, Z.M curated and analysed the Chengdu cohort. E.B. carried out *in silico* analysis. V.G.B., Z.M. and X.X. acquired funding and V.G.B. and J.E. provided supervision. V.G.B. Z.M. E.B. drafted the manuscript. All authors read and commented on the manuscript.

## Funding

This work was funded by an Endeavour Scholarship (734/2018/878) to Z.M.; Cancer Research UK (C157/A23219) to L.G.‐C. and (CANTAC721\100018) to E.B.; Sichuan Science and Technology Program (NO. 2022YFS0601) and Sichuan Provincial People's Hospital (NO. 2021ZX02) to X.X. and was supported by the Cancer Research UK Scotland Centre (CTRQQR‐2021\100006).

## Ethics Statement

Tumours were collected under the approval of the research ethics committee of the West of Scotland Research Ethics Service REC4 (REC reference: 22/WS/0020, IRAS project ID: 306447). All material used within this work was obtained from the Pathology Diagnostic Archives, and as such is unconsented surplus rather than project‐specific material. The unconsented use of the samples used for this study follows the Human Tissue Authority (HTA) legislation on consent exemption and is covered under the National Health Service (NHS) of Greater Glasgow and Clyde (GGC) Biorepository consent exemption policy.

## Conflicts of Interest

The authors declare no conflicts of interest.

## Supporting information


**Figure S1:** Formation of PAPP‐A cohort.
**Figure S2:** Association between total PAPP‐A expression and CSS in.
**Figure S3:** Association between tumour and stromal PAPP‐A expression.
**Table S1:** Survival analysis for total PAPP‐A and other prognostic.
**Table S2:** Survival analysis for stromal PAPP‐A and other prognostic.
**Table S3:** Association between total PAPP‐A expression and clinical.
**Table S4:** Association between stromal PAPP‐A expression and clinical.
**Table S5:** Survival analysis for total PAPP‐A and other prognostic.
**Table S6:** Survival analysis for stromal PAPP‐A and other prognostic.

## Data Availability

The clinical data that support the findings of this study are available via the NHS Greater Glasgow and Clyde Safe Haven (GSH/18/ON008 and GSH/21/ON/008).
