# Supplementary material for: High Expression of PAPP‐A Predicts Poor Outcomes in Oestrogen Receptor‐Positive Breast Cancer Patients
Source: Cancer Med. 2026 Apr 13;15(4):e71815. doi: 10.1002/cam4.71815 (PMC13072045; doi:10.1002/cam4.71815)
Supplement: Supplementary file 1 — Figure S1: Formation of PAPP‐A cohort. Figure S2: Association between total PAPP‐A expression and CSS in. Figure S3: Association between tumour and stromal PAPP‐A expression. Table S1: Survival analysis for total PAPP‐A and other prognostic. Table S2: Survival analysis for stromal PAPP‐A and other prognostic. Table S3: Association between total PAPP‐A expression and clinical. Table S4: Association between stromal PAPP‐A expression and clinical. Table S5: Survival analysis for total PAPP‐A and other prognostic. Table S6: Survival analysis for stromal PAPP‐A and other prognostic. [file CAM4-15-e71815-s001.pdf]

High expression of PAPP-A predicts poor outcomes in ER-positive breast cancer

Zeanap Mabruk, Esme Bullock, Xue Xiao, Jingjing Guo, Xuan Zhu, Laura Gómez-Cuadrado, Claus Oxvig, Elizabeth Mallon, Aula Ammar, Amna Malty, Kathryn Pennel, Joanne Edwards, Valerie G Brunton

Supplementary Information

This file contains:

Supplementary Methods

Supplementary Figure S1: Formation of PAPP-A cohort.

Supplementary Figure S2: Association between total PAPP-A expression and CSS in breast cancer molecular subtypes.

Supplementary Figure S3: Association between tumour and stromal PAPP-A expression and CSS.

Supplementary Table S1: Survival analysis for total PAPP-A and other prognostic factors.

Supplementary Table S2: Survival analysis for stromal PAPP-A and other prognostic factors.

Supplementary Table S3: Association between total PAPP-A expression and clinical factors.

Supplementary Table S4: Association between stromal PAPP-A expression and clinical factors.

Supplementary Table S5: Survival analysis for total PAPP-A and other prognostic factors in ILC.

Supplementary Table S6: Survival analysis for stromal PAPP-A and other prognostic factors in ILC.

Supplementary Methods

Clinical cohorts

Glasgow cohort of 246 human primary operable ILC: The primary tumour was available as formalin fixed paraffin-embedded (FFPE) blocks for evaluation. A database including clinicopathological information, adjuvant treatments, recurrence, and survival data was available from Glasgow safe haven (safe haven number: GSH/21/ON/008). ER, PR and HER2 status had been carried out retrospectively in diagnostic labs to ensure standardisation of techniques. Nottingham grading system was applied to grade tumours. The tumour microarray (TMA) for this cohort was constructed by the Glasgow Tissue Research Facility (University of Glasgow). Briefly, FFPE was obtained from pathology services, and full sections were cut, hematoxylin and eosin (H&E) stained and marked up by pathologist Prof Elizabeth Mallon to pick tumour rich regions. Three 0.6 mm cores were selected from each block and embedded into three separate paraffin blocks. Cores from other tissue types were also embedded on the same block as a positive control during staining. TMA maps were created in such a way that each core could be uniquely identified by a TMA-ID, anonymously connecting it to the information stored in the cohort database.

Chengdu cohort of 172 ER+ treatment naïve breast cancers: The primary tumour was available as formalin fixed paraffin-embedded (FFPE) blocks for evaluation. H&E staining and ER, PR, HER2 status had been carried out retrospectively in diagnostic labs. Immunohistochemistry staining of PAPP-A together with original pathology reports were then reviewed by two senior pathologists to reconfirm pathological diagnoses.

Supplementary Figure S1

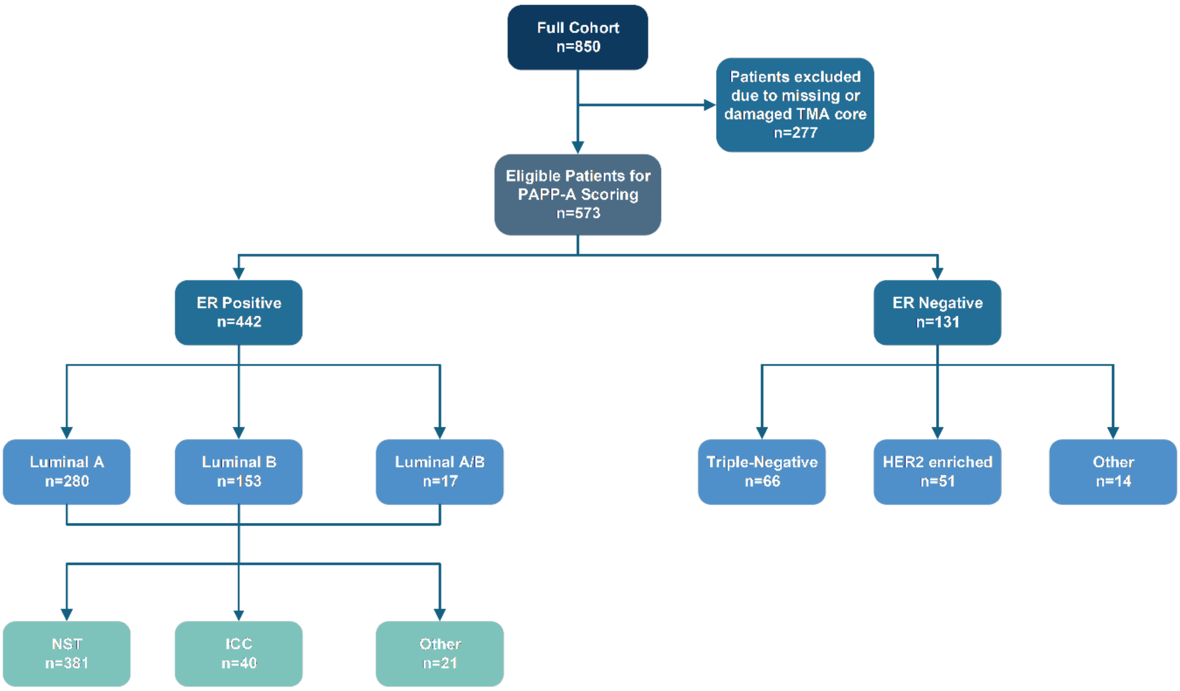

Figure S1. Formation of PAPP-A cohort. Flow diagram to illustrate the number of patients within the Glasgow cohort with at least one core which was assessable for PAPP-A expression in tumour and stroma. The numbers available for subgroup analysis by ER status, and molecular subtypes are also shown. Missing Ki67 and HER2 immunohistochemistry data for 17 ER positive tumours (termed Luminal A/B) prevented their classification as either Luminal A or Luminal B and were omitted from subtype-specific analyses.

Supplementary Figure S2

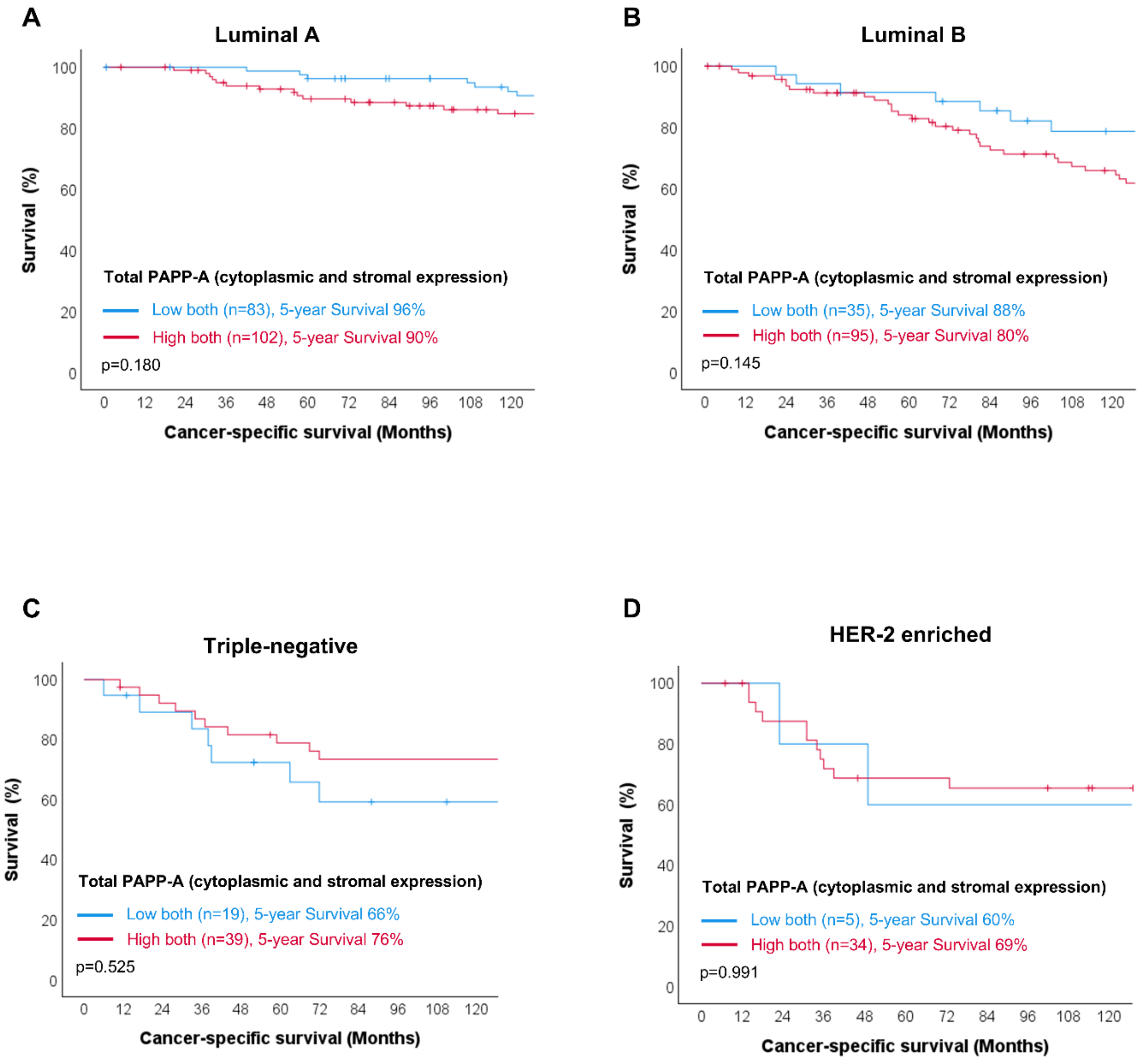

Figure S2. Association between total PAPP-A expression and CSS in breast cancer molecular subtypes. Kaplan Meier curve showing the association between the expression of total PAPP-A and CSS across breast cancer molecular subtypes.

Supplementary Figure S3

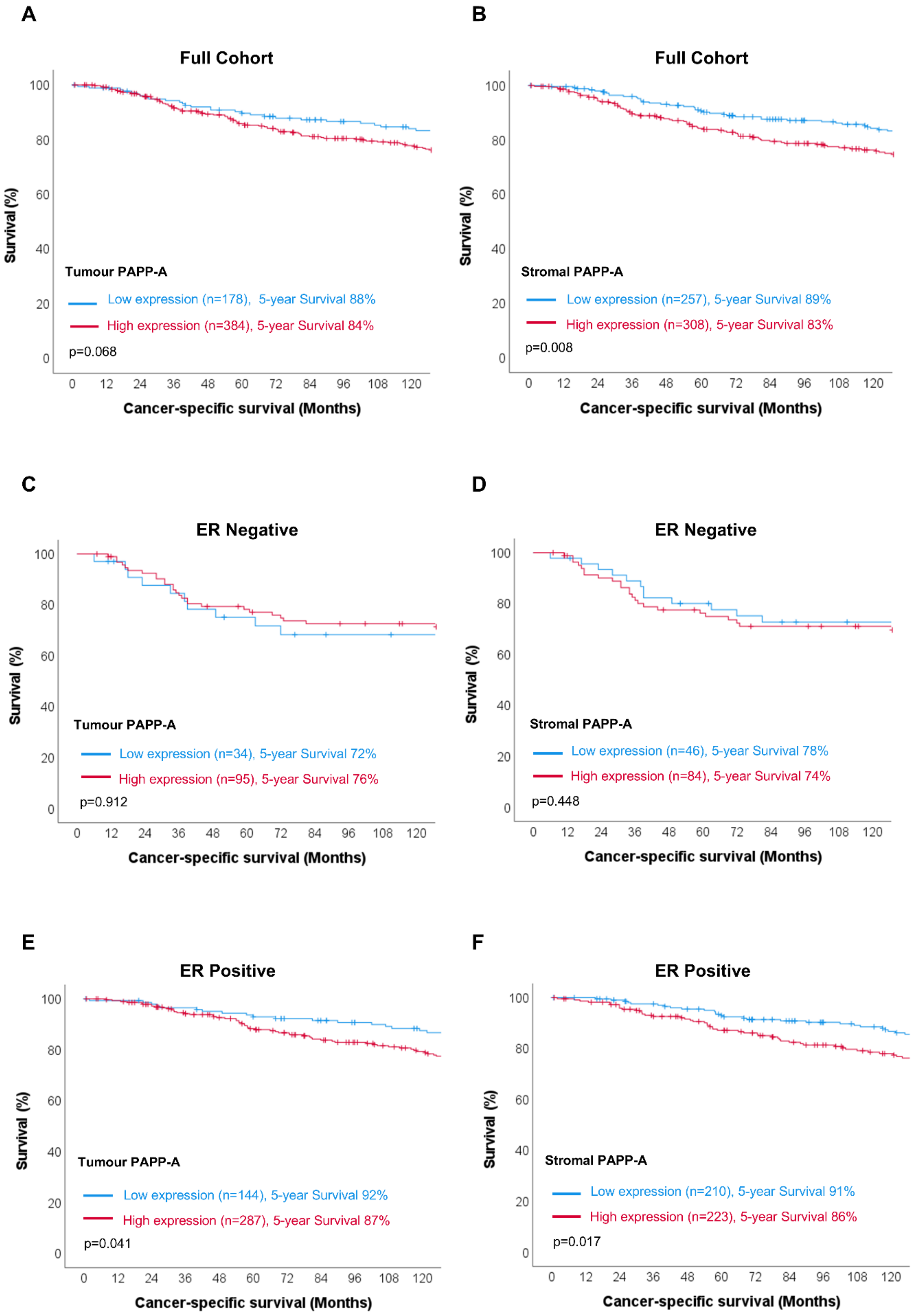

Figure S3. Association between tumour and stromal PAPP-A expression and CSS. Kaplan Meier curves showing the association between tumour PAPP-A expression and CSS across full cohort (A), in ER-negative (C), and ER-positive patients (E), and stromal PAPP-A across full cohort (B), in ER-negative (D), and ER-positive patients (F).

Supplementary Table S1

	Univariate analysis Total PAPP-A		Multivariate analysis Total PAPP-A	
	**HR (95%CI)**	**p value**	**HR (95%CI)**	**p value**
**Age**				
<50yrs	1	0.748	–	–
>50yrs	0.949 (0.689-1.307)			
**Tumour size**				
<20mm	1	**<0.001**	1	**0.015**
21-49mm	2.191 (1.593-3.015)	**<0.001**	1.447 (0.929-2.254)	0.103
>50mm	4.460 (2.708-7.346)	**<0.001**	2.644 (1.356-5.154)	**0.004**
**Grade**				
I	1	**<0.001**	–	0.535
II	2.017 (1.134-3.589)	**0.017**		0.473
III	3.949 (2.251-6.928)	**<0.001**		0.287
**Nodal status**				
Negative	1	**<0.001**	1	**<0.001**
Positive	3.246 (2.369-4.446)		2.442 (1.583-3.767)	
**ER status**				
Negative	1	**<0.001**	–	0.383
Positive	0.567 (0.420-0.765)			
**PR status**				
Negative	1	**<0.001**	–	0.564
Positive	0.538 (0.393-0.737)			
**HER2 status**				
Negative	1	**0.001**	–	0.890
Positive	1.835 (1.284-2.623)			
**Molecular subtype**				
Luminal A	1	**<0.001**	1	**<0.001**
Luminal B	2.293 (1.533-3.429)	**<0.001**	2.668 (1.588-4.483)	**<0.001**
Triple negative	2.544 (1.708-3.789)	**<0.001**	3.090 (1.690-5.652)	**<0.001**
HER2-enriched	3.054 (1.867-4.995)	**<0.001**	3.551 (1.813-6.954)	**<0.001**
**Ki67**				
<15%	1	**0.002**	–	0.666
>15%	1.642 (1.198-2.250)			
**Total PAPP-A**				
Low	1	**0.027**	–	0.166
High	1.658 (1.058-2.597)			

Table S1. Survival analysis for total PAPP-A and other prognostic factors. Univariate and multivariate Cox regression analysis of the relationship between total PAPP-A, other known prognostic clinicopathological factors and CSS. Factors with a significance of $p<0.05$ on univariate analysis were entered into the multivariate analysis.

Supplementary Table S2

	Univariate analysis Stromal PAPP-A		Multivariate analysis Stromal PAPP-A	
	**HR (95% CI)**	**p value**	**HR (95%CI)**	**p value**
**Age**				
<50yrs	1	0.748	–	–
>50yrs	0.949 (0.689-1.307)			
**Tumour size**				
<20mm	1	**<0.001**	1	**0.003**
21-49mm	2.191 (1.593-3.015)	**<0.001**	1.466 (0.978-2.198)	0.064
>50mm	4.460 (2.708-7.346)	**<0.001**	2.877 (1.545-5.358)	**<0.001**
**Grade**				
I	1	**<0.001**	1	0.097
II	2.017 (1.134-3.589)	**0.017**	1.352 (0.675-2.707)	0.395
III	3.949 (2.251-6.928)	**<0.001**	1.982 (0.953-4.122)	0.067
**Nodal status**				
Negative	1	**<0.001**	1	**<0.001**
Positive	3.246 (2.369-4.446)		2.400 (1.613-3.570)	
**ER status**				
Negative	1	**<0.001**	–	0.282
Positive	0.567 (0.420-0.765)			
**PR status**				
Negative	1	**<0.001**	–	0.585
Positive	0.538 (0.393-0.737)			
**HER2 status**				
Negative	1	**0.001**	–	0.774
Positive	1.835 (1.284-2.623)			
**Molecular subtype**				
Luminal A	1	**<0.001**	1	
Luminal B	2.293 (1.533-3.429)	**<0.001**	1.878 (1.164-3.032)	**0.027**
Triple negative	2.544 (1.708-3.789)	**<0.001**	2.072 (1.138-3.770)	**0.010**
HER2-enriched	3.054 (1.867-4.995)	**<0.001**	2.202 (1.165-4.162)	**0.017**
				**0.015**
**Ki67**				
<15%	1	**0.002**	–	0.751
>15%	1.642 (1.198-2.250)			
**Stromal PAPP-A**				
Low	1	**0.008**	–	0.141
High	1.676 (1.147-2.451)			

Table S2. Survival analysis for stromal PAPP-A and other prognostic factors. Univariate and multivariate Cox regression analysis of the relationship between stromal PAPP-A, other known prognostic clinicopathological factors and CSS. Factors with a significance of $p<0.05$ on univariate analysis were entered into the multivariate analysis.

Supplementary Table S3

	Total PAPP-A		P value
	Low<br>n (%)	High<br>n (%)	
**Age**			
<50yrs	43 (28.3)	88 (31.4)	0.497
>50yrs	109 (71.7)	192 (68.6)	
**Tumour size**			
<20mm	93 (61.2)	143 (51.1)	0.169
21-49mm	47 (30.9)	119 (42.5)	
>50mm	12 (7.9)	18 (6.4)	
**Grade**			
I	40 (26.3)	39 (13.9)	**<0.001**
II	82 (53.9)	123 (43.9)	
III	30 (19.7)	118 (42.1)	
**Nodal status**			
Negative	89 (58.9)	152 (55.3)	0.465
Positive	62 (41.1)	123 (44.7)	
**ER status**			
Negative	30 (19.7)	80 (28.7)	**0.039**
Positive	122 (80.3)	199 (71.3)	
**PR status**			
Negative	60 (39.5)	144 (51.6)	**0.016**
Positive	92 (60.5)	135 (48.4)	
**HER2 status**			
Negative	132 (89.2)	209 (76.0)	**<0.001**
Positive	16 (10.8)	66 (24.0)	
**Molecular subtype**			
Luminal A	86 (58.9)	104 (38.2)	**<0.001**
Luminal B	36 (24.7)	95 (34.9)	
Triple-negative	19 (13)	39 (14.3)	
HER2-enriched	5 (3.4)	34 (12.5)	
**Ki67**			
<15%	108 (72.5)	155 (57)	**0.001**
>15%	41 (27.5)	117 (43)	

Table S3. Association between total PAPP-A expression and clinical factors. Chi-squared test for associations between total PAPP-A and other clinicopathological factors.

Supplementary Table S4

	Stromal PAPP-A		P value
	**Low** n (%)	**High** n (%)	
**Age**			
<50yrs	77 (29.2)	92 (29.6)	0.913
>50yrs	187 (70.8)	219 (70.4)	
**Tumour size**			
<20mm	164 (62.1)	156 (50.2)	**0.016**
21-49mm	84 (31.8)	135 (43.4)	
>50mm	16 (6.1)	20 (6.4)	
**Grade**			
I	72 (27.3)	42 (13.5)	**<0.001**
II	133 (50.4)	146 (46.9)	
III	59 (22.3)	123 (39.5)	
**Nodal status**			
Negative	158 (60.5)	165 (53.9)	0.133
Positive	103 (39.5)	141 (46.1)	
**ER status**			
Negative	46 (17.5)	85 (27.4)	**0.004**
Positive	217 (82.5)	225 (72.6)	
**PR status**			
Negative	100 (38)	156 (50.3)	**0.003**
Positive	163 (62)	154 (49.7)	
**HER2 status**			
Negative	227 (87.6)	239 (78.1)	**0.003**
Positive	32 (12.4)	67 (21.9)	
**Molecular subtype**			
Luminal A	155 (62)	125 (41.4)	**<0.001**
Luminal B	56 (22.4)	99 (32.8)	
Triple-negative	23 (9.2)	43 (14.2)	
HER2-enriched	16 (6.4)	35 (11.6)	
**Ki67**			
<15%	188 (74.3)	180 (59.6)	**<0.001**
>15%	65 (25.7)	122 (40.4)	
**Tumour PAPP-A**			
Low	152 (58.0)	30 (9.7)	**<0.001**
High	110 (42.0)	280 (90.3)	

Table S4. Association between stromal PAPP-A expression and clinical factors. Chi-squared test for associations between stromal PAPP-A and other clinicopathological factors.

Supplementary Table S5

	Univariate analysis Total PAPP-A		Multivariate analysis Total PAPP-A	
	HR (95%CI)	p value	HR (95%CI)	p value
**Recurrence**				
No recurrence	1	**<0.001**	1	**<0.001**
Local	4.447 (2.028-9.754)	**<0.001**	7.837 (2.671-22.989)	**<0.001**
Distant	10.855 (5.997-19.648)	**<0.001**	6.519 (1.413-30.067)	**0.016**
**Tumour size**				
≤2cm	1	**0.037**	–	0.580
2.1-5cm	0.781 (0.442-1.378)	0.394		0.743
5cm	2.386 (1.034-5.505)	**0.042**		0.414
**Screen detected**				
No	1	**0.006**	–	0.150
Yes	0.439 (0.245-0.786)			
**HER2 status**				
Negative	1	0.618	–	–
Positive	1.347 (0.419-4.329)			
**Nodal status**				
Negative	1	**0.047**	–	0.857
Positive	1.724 (1.007-2.952)			
**Lymph invasion**				
No	1	0.527	–	–
Yes	0.720 (0.260-1.993)			
**Neoadjuvant therapy**				
No	1	0.211	–	–
Yes	1.719 (0.735-4.018)			
**Total PAPP-A**				
Low	1	**0.019**	–	0.160
High	3.115 (1.207-8.043)			

Table S5. Survival analysis for total PAPP-A and other prognostic factors in ILC. Univariate and multivariate Cox regression analysis of the relationship between stromal PAPP-A, other known prognostic clinicopathological factors and CSS. Factors with a significance of $p<0.05$ on univariate analysis were entered into the multivariate analysis.

Supplementary Table S6

	Univariate analysis Stromal PAPP-A		Multivariate analysis Stromal PAPP-A	
	HR (95% CI)	p value	HR (95% CI)	p value
**Recurrence**				
No recurrence	1	**<0.001**	1	**<0.001**
Local	4.447 (2.028-9.754)	**<0.001**	4.490 (1.879-10.733)	**<0.001**
Distant	10.855 (5.997-19.648)	**<0.001**	7.467 (3.567-15.628)	**<0.001**
**Tumour size**				
≤2cm	1	**0.037**		0.479
2.1-5cm	0.781 (0.442-1.378)	0.394	_	0.227
≥5cm	2.386 (1.034-5.505)	**0.042**		0.644
**Screen detected**				
No	1	**0.006**	1	0.080
Yes	0.439 (0.245-0.786)		0.512 (0.242-1.083)	
**HER2 status**				
Negative	1	0.618	_	_
Positive	1.347 (0.419-4.329)			
**Nodal status**				
Negative	1	**0.047**	_	0.716
Positive	1.724 (1.007-2.952)			
**Lymph invasion**				
No	1	0.527	_	_
Yes	0.720 (0.260-1.993)			
**Neoadjuvant therapy**				
No	1	0.211	_	_
Yes	0.211 (0.735-4.018)			
**Stromal PAPP-A**				
Low	1	**0.035**	1	0.087
High	2.005 (1.049-3.833)		1.817 (0.918-3.597)	

Table S6. Survival analysis for stromal PAPP-A and other prognostic factors in ILC. Univariate and multivariate Cox regression analysis of the relationship between stromal PAPP-A, other known prognostic clinicopathological factors and CSS. Factors with a significance of $p<0.05$ on univariate analysis were entered into the multivariate analysis.
